# The association between uncontrolled hyperglycemia (Type-2 Diabetes) and cardiovascular sequelae in patients with and without insulin

**DOI:** 10.1016/j.jsps.2024.102168

**Published:** 2024-09-04

**Authors:** Sultan Alghadeer, Abdullah M. Mubarak, Bashayr Alsuwayni, Faisal Almurdhi, Hazim Almalki, Metib Alotaibi

**Affiliations:** a Department of Clinical Pharmacy, College of Pharmacy, King Saud University, Riyadh, Saudi Arabia; b Department of Basic Sciences, PSCEMS, King Saud University, Riyadh, Saudi Arabia; c Corporate of Pharmacy Services, King Saud University Medical City, Riyadh, Saudi Arabia; d Diabetes Center, King Saud University Medical City, Riyadh, Saudi Arabia

**Keywords:** Cardiovascular, Complications, Diabetes, Hyperglycemia, Saudi Arabia, Uncontrolled, Insulin

## Abstract

**Introduction:**

Despite the availability of new cardio-protective oral hypoglycemic drugs, insulin is often recommended as an add-on therapy for type-2 diabetes with hemoglobin A1C (HbA1C) ≥ 9. Introducing insulin as a choice for patients with uncontrolled hyperglycemia (HbA1C≥9) has been questionably associated with cardiovascular sequelae. This study aims to examine the association between insulin use and cardiovascular effects in type-2 diabetic patients with uncontrolled hyperglycemia.

**Methodology:**

A retrospective observational cohort study was conducted to identify cardiovascular complications between the two groups (patients with HbA1C≥9% on insulin versus those with HbA1C≥9% without insulin) at King Saud University Medical City (KSUMC). Patients with type-2 diabetes whose HbA1C was ≥ 9 during the period from 2015 to 2018 and who were followed up within the hospital for at least 5 years until the end of 2022 were included in the study.

**Results:**

A total of 366 patients were included in the study; 286 patients were on insulin, while 80 patients were not. The median baseline HbA1C levels were comparable between the two groups (10.2 versus 9.8). After 5 years of follow-up, there was no significant difference between the groups (29.4 % of insulin users versus 18.8 % of non-insulin users; p = 0.065). However, the incidence of other diabetes complications, such as retinopathy, nephropathy, and neuropathy, was significantly higher among patients who were on insulin compared to those not on insulin (50.7 % versus 27.5 %; p = 0.005). Additionally, the average of the last three HbA1C readings and the overall average HbA1C readings were significantly higher among patients who were on insulin (9.67 % versus 9.07 %; p = 0.001) compared to those not on insulin (9.64 % versus 9.11 %; p = 0.005).

**Conclusion:**

Our study did not find a significant association between the use of insulin and cardiovascular complications. The association between insulin therapy and the development of other diabetes complications warrants further investigation.

## 1 Introduction

There are two types of diabetes: type 1 diabetes, an autoimmune disease that destroys pancreatic beta cells, necessitating lifelong insulin use, and type 2 diabetes, the most common type, which can result from insulin resistance, insulin secretion deficiency, or both.(Banday et al., 2020, Dean et al., 2004) Long-term exposure to diabetes and elevated blood glucose levels can lead to microvascular and macrovascular diseases and premature death.(Dal Canto et al., 2019) In Saudi Arabia, diabetes is one of the most common chronic diseases. In 2016, the World Health Organization estimated that around 7 million people in the population are diabetic, and around 3 million are prediabetic.(Al Dawish et al., 2016) Effective diabetes management and identifying the best treatment options are crucial.

In the past, insulin was considered the last step in the therapeutic plan for patients with type-2 diabetes mellitus (type-2 DM).(Swinnen et al., 2009) The potential disadvantages of lowering blood glucose, causing pain, increasing weight, and requiring strict adherence to therapy often delay the introduction of insulin for type-2 DM.(Nakar et al., 2007, Polonsky et al., 2005) However, this approach has changed. Early initiation of insulin therapy is now advocated if hemoglobin A1C (HbA1C) remains above 7 % for 2 to 3 months despite dual oral therapy.(Nathan DM, Buse JB, Davidson MB, Ferrannini E, HolmanRR, Sherwin R, Zinman B. Medical management of hyperglycemia in type 2 diabetes: a consensus algorithm for the initiation and adjustment of therapy: a consensus statement of the American Diabetes Association and the European Association for the Study of Diabetes. Diabetes Care., 2009, Houlden et al., 2007) The probable avoidance of macro- and micro-vascular complications due to high blood glucose and the confirmed enhancement of bodily-secreted insulin have prompted the early addition of hypoglycemic drugs like insulin.(Brown et al., 2004, Weng et al., 2008) However, the threshold for HbA1C to start insulin therapy for type-2 DM is not fully defined.

The 2022 American Diabetes Association (ADA) and European Association for the Study of Diabetes (EASD) guidelines for managing type-2 DM emphasize an individualized approach.(Davies et al., 2022) They recommend using insulin therapy in combination with other hypoglycemic agents such as metformin, sodium-glucose co-transporter 2 inhibitors (SGLT2i), or glucagon-like peptide 1 receptor agonists (GLP-1 RA) after a failed trial. They also recommend insulin as initial therapy if the patient has highly elevated blood glucose, defined as HbA1C≥10 %. Similarly, in 2018, the ADA suggested using insulin for type-2 DM as an initial or dual therapy if HbA1C≥10 and as initial dual therapy if HbA1C≥9. (American Diabetes Association, 2018).

Some studies have shown an association between high insulin levels and an increased risk of cardiovascular disease. (Ruige et al., 1998) Insulin is associated with worsening heart failure in patients with heart failure with reduced ejection fraction compared to non-insulin treatment. (Cosmi et al., 2018) In a randomized trial studying insulin degludec and glargine, serious side effects were reported by approximately 16 % of the subjects, with cardiac disorders being the most serious adverse effect. (Rodbard et al., 2013) One article indicates that some large studies demonstrate serious safety issues with insulin, including cardiovascular events. (Schwartz et al., 2016).

High HbA1C levels are associated with an increased risk of macrovascular events and mortality. (Laiteerapong et al., 2019) Additionally, reducing HbA1C to less than 9 % is associated with positive cardiovascular outcomes based on ADA guidelines, which recommend adding insulin as a choice for symptomatic diabetic patients with HbA1C>9. (Davies et al., 2022, American Diabetes Association, 2018) However, the use of insulin for patients with uncontrolled diabetes has been associated with negative cardiovascular outcomes compared to other anti-diabetic medications. Therefore, this study aims to examine the association between insulin use and cardiovascular effects in type-2 DM patients with uncontrolled hyperglycemia.

## 2 Methods

A retrospective observational cohort study was conducted to detect the difference in cardiovascular effects between insulin users and non-insulin users in type-2 DM patients with uncontrolled hyperglycemia. The study participants’ data were obtained from King Saud University Medical City (KSUMC), Riyadh, Saudi Arabia. The study was ethically approved by the institutional review board at KSUMC (research project No. E-22–6978).

All adult patients diagnosed with type-2 DM whose HbA1C≥9 during the period from 2015 to 2018 and who were followed up within the hospital for at least 5 years until the end of 2022 were included in the study. The patients were then stratified based on insulin usage into two groups: group-1 (uncontrolled type-2 DM with HbA1C≥9 started on insulin) versus group-2 (uncontrolled type-2 DM with HbA1C≥9 without insulin use throughout the study period). Any patient with incomplete initial or follow-up data, less than 5 years of follow-up, or with baseline macro- or micro-complications of diabetes was excluded from the study. The primary outcome was to detect cardiovascular complications such as coronary artery disease, stroke, hypertension, heart failure, or other cardiovascular diseases. The secondary outcomes investigated other diabetes complications such as diabetic nephropathy, neuropathy, retinopathy, peripheral artery disease, or diabetic foot complications.

Data collected during the study included demographic information (age, gender, body mass index), past medical history, baseline and follow-up laboratory data such as HbA1C, fasting blood glucose, serum creatinine, lipid panel, baseline and follow-up vital signs readings, insulin data (start date, type, dose and frequency, changes of doses), other hypoglycemic medications, cardiovascular, and other diabetes complications.

Descriptive analysis with counts and percentages was performed to express the categorical variables. Kolmogorov-Smirnov and Shapiro-Wilk tests were utilized to test for normal distribution, with median and interquartile range (IQR) expressed for continuous variables. Non-parametric Mann-Whitney *U* test and Chi-square test were used to compare the two observed numerical groups and to assess the association of the categorical variables, respectively. However, these two tests do not control for confounding variables. All statistical analyses were conducted using SPSS (SPSS Inc., Chicago, IL, USA) software.

## 3 Results

A total of 366 patients were included in the study; 286 patients were on insulin, while 80 patients were not. In both groups, most patients were either overweight or obese. Female gender constituted the majority of the insulin-reliant group (56.3 %), while male gender constituted the majority of the non-insulin-reliant group (61.3 %). The median baseline HbA1C levels were comparable between the two groups (10.2 versus 9.8). The baselines of blood glucose (BG) levels, serum creatinine (SCr), number of related tests (HbA1C, BG, and SCr), and presence of other comorbidities were also comparable. Details of demographic characteristics are listed in Table 1.

**Table 1:** Baseline characteristics (n = 366).

**Characteristic**	**On Insulin** **N=286 (78.1 %)**	**Not on Insulin** **N=80 (21.9 %)**
**Gender**		
Male	125 (43.7 %)	49 (61.3 %)
Female	161 (56.3 %)	31 (38.8 %)
**Age (Years), (median, IQR)**	66 (13)	66.5 (13.75)
**Average BMI Classification**		
Below 18.5 underweight	1 (0.3 %)	0 (0 %)
18.5–24.9 normal	28 (9.8 %)	14 (17.5 %)
25–29.9 Over weight	83 (29 %)	33 (41.2 %)
Over 30 Obesity	174 (60.8 %)	33 (41.2 %)
**Baseline HbA1C (median, IQR)**	10.2 (1.9)	9.8 (1.4)
**Number of HbA1C tests (median, IQR)**	12 (7)	10.5 (5)
**Baseline SCr (median, IQR) in mmol/L**	73 (29)	69 (29)
**Number of SCr tests (median, IQR)**	14 (12)	12 (8)
**BG baseline (median, IQR) in mmol/L**	10.97 (5.28)	11.5 (5.19)
**Number of BG**	13.5 (11)	11 (6)
**Other Diabetic Medications**		
Biguanides (Metformin)	228	62
Sulfonylureas	53	77
TZDs	19	12
α-glucosidase inhibitors	7	3
Meglitinides	0	4
DPP-4 inhibitors	216	57
GLP-1 RA	59	7
SGLT2i	15	3
Comorbidities *		
Dyslipidaemia	264 (92.3 %)	70 (87.5 %)
HTN	251 (87.8 %)	65 (81.2 %)
Smoking	18 (6.3 %)	5 (6.2 %)

BG: blood glucose; BMI: body mass index; DPP-4: Dipeptidyl Peptidase-IV; GLP-1 RA: Glucagon-like peptide-1receptor agonists; HbA1c: hemoglobin A1C; Scr: serum creatinine; SGLT2i: Sodium-glucose cotransporter-2 inhibitors; TZDs: Thiazolidinediones.

**Fig. 1:**
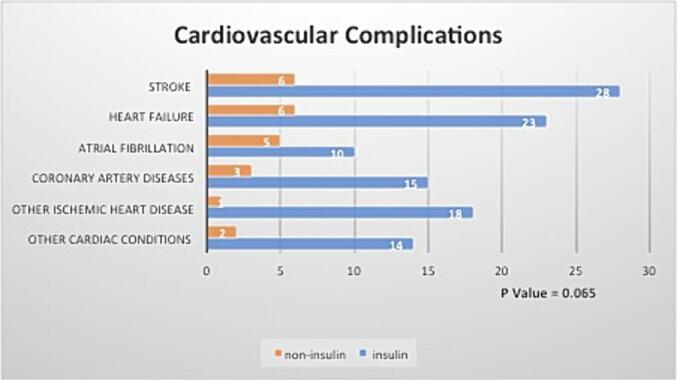
Cardiovascular complications.

The cardiovascular complications after 5-years of follow-up showed no significant difference in cardiovascular complications between the groups (29.4 % of cardiovascular events for patients with uncontrolled type-2 DM with HbA1C≥9 on insulin versus 18.8 % of cardiovascular events for those not on insulin; p = 0.065) (Table 2). Stroke and heart failure were the most common cardiovascular sequelae in both groups. The type and distribution of cardiovascular events are illustrated in Fig. 1.

**Table 2:** Primary and secondary outcomes (n = 366)*.

**Outcome**	**On Insulin** **N=286 (78.1 %)**	**Not on Insulin** **N=80 (21.9 %)**	**P value**
**Cardiovascular Complications**	84 (29.4 %)	15 (18.8 %)	0.065
**DM Complications**	145 (50.7 %)	22 (27.5 %)	0.005
**Average of last 3 HbA1C readings (median, IQR)**	9.67 (1.51)	9.07 (1.59)	0.001
**Average HbA1C (median, IQR)**	9.64 (1.45)	9.11 (1.76)	0.005
**Average SCr (median, IQR) in mmol/L**	77.56 (33.26)	71.57 (30.21)	0.088
**Average BG (median, IQR) in mmol/L**	10.47 (3.01)	10 (3.16)	0.137

*Chi-square and Mann-Whitney *U* test.

BG: blood glucose; DM: diabetes; HbA1c: hemoglobin A1C; Scr: serum creatinine.

**Fig. 2:**
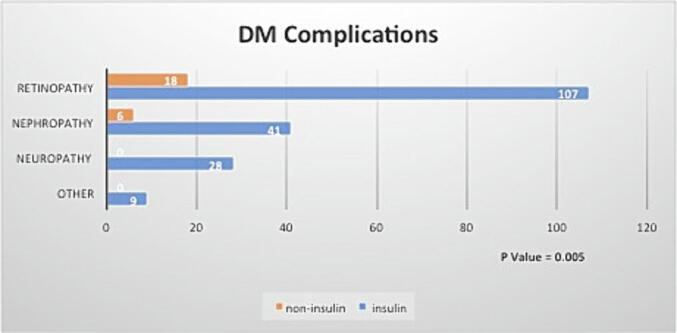
Diabetes complications.

In contrast to cardiovascular complications, the incidence of diabetes complications such as retinopathy, nephropathy, and neuropathy was significantly higher among patients on insulin compared to those not on insulin (50.7 % versus 27.5 %; p = 0.005). Additionally, the average of the last three HbA1C readings and the average HbA1C readings were significantly higher among patients who were on insulin (9.67 % versus 9.07 %; p = 0.001) compared to those not on insulin (9.64 % versus 9.11 %; p = 0.005). There was no significant difference in cardiovascular complications between the two groups in terms of average SCr and BG levels (Table 2). Retinopathy, followed by nephropathy, was the most common diabetes complication in both groups (Fig. 2).

Despite a slight decrease in total cholesterol and LDL levels and an increase in HDL levels in both groups, these differences were not significant. Patients’ lipid profile readings are shown in Table 3.

**Table 3:** Patients’ lipid profile (n = 366)*.

**Outcome**	**On Insulin** **N=286 (78.1 %)**	**Not on Insulin** **N=80 (21.9 %)**	**P value**
**Average of total Cholesterol last reading (median, IQR) in mmol/L**	4.20 (6.02)	4.24 (1.22)	0.453
**Average of total Cholesterol difference reading (median, IQR) in mmol/L**	−0.27 (1.39)	−0.34 (1.2)	0.238
**Average of LDL Last reading (median, IQR) in mmol/L**	2.13 (1.13)	2.25 (1.04)	0.540
**Average of LDL difference reading (median, IQR) in mmol/L**	−0.29 (1.19)	−0.32 (1.12)	0.539
**Average of HDL Last reading (median, IQR) in mmol/L**	1.14 (0.41)	1.2 (0.47)	0.723
**Average of HDL difference reading (median, IQR) in mmol/L**	0.07 (0.3)	0.11 (0.27)	0.304

*Mann-Whitney *U* test.

HDL: High-density lipoprotein; LDL: low-density lipoprotein

Out of 366 patients, 273 used dipeptidyl peptidase-4 (DPP-4) inhibitors (216 were on insulin while 57 were not). The incidence of retinopathy was 35.9 % (n = 98/273) among DPP-4 inhibitor users compared to 29 % (n = 27/98) among non-DPP-4 inhibitor users (p = 0.204).

## 4 Discussion

Insulin therapy is common, and the association between insulin use in patients with uncontrolled type II diabetes mellitus, defined as having an HbA1C of more than 9 %, and cardiovascular sequelae is questionable. Some studies show a weak association between insulin levels and cardiovascular complications. (Ruige et al., 1998) These studies, reported in *meta*-analyses, found that the relative risk (RR) for cardiovascular complications is 1.18 (95 % CI 1.081.29) for a 50 pmol/l increase in fasting insulin. Another study comparing the efficacy and safety of two types of insulin (degludec versus glargine) in type-2 DM patients found that approximately 15–16 % of patients reported serious side effects, most of which were cardiac disorders.(Rodbard et al., 2013) However, a high HbA1C level is considered a cofactor that may affect this association, as higher HbA1C levels alone are associated with an increased risk of macrovascular events and mortality. (Laiteerapong et al., 2019).

In our study, with a sample of 99 patients experiencing cardiovascular complications, 84 were on insulin therapy, and 15 were not (p = 0.065). This study presents a non-significant association between insulin therapy and cardiovascular complications. Among insulin users, stroke was the most prevalent cardiovascular complication (28 events), followed by heart failure (23 events). The connection between stroke and insulin therapy, a novel aspect, is discussed, noting it may be linked to either insulin therapy or uncontrolled diabetes. (Garg et al., 2006) Additionally, coronary artery disease (CAD) and atrial fibrillation (AF) were identified in 15 and 10 insulin users, respectively, while other ischemic heart diseases (IHD) and various cardiovascular complications occurred in 18 and 14 events, respectively.

When comparing our findings to a study investigating the relationship between insulin treatment and the risk of major adverse cardiovascular events (MACE) in stable type 2 diabetes patients, (Schwartz et al., 2016) especially those with recent acute coronary syndrome (ACS). An analysis utilizing data from the BETonMACE phase 3 trial that compared incidence of MACE among type-2 DM patients with ACS who received apabetalone versus placebo revealed that despite receiving evidence-based therapies, insulin-treated patients (n = 829) have a significantly higher MACE risk (20.4 % in insulin users versus 12.8 % in non-insulin users; p = 0.0001), which persists even after adjustments for several variables (HR 2.10; p = 0.0002). The thiazolidinedione class used for managing type-2 DM is also associated with an increased risk of heart failure. (Chaggar et al., 2009) Three out of the twenty-three patients on insulin therapy who had heart failure were using pioglitazone, a thiazolidinedione agent. This might influence the incidence of heart failure in our study sample.

A study conducted at Jordan University Hospital aimed to identify risk factors associated with diabetic retinopathy in more than 900 diabetic patients. Out of 1961 eyes examined, 64.1 % exhibited diabetic retinopathy, with 54.8 % having nonproliferative diabetic retinopathy (NPDR), 9.3 % having proliferative diabetic retinopathy (PDR), and 30.8 % having maculopathy. Their analysis revealed that old age, long diabetes duration, poor glycemic control, uncontrolled blood pressure, and nephropathy were significantly associated with diabetic retinopathy. Additionally, maculopathy was linked to hypertension, proteinuria, and high cholesterol. However, the risk was not linked to the type of medication management received. In contrast, a study conducted in Saudi Arabia to investigate the incidence of diabetic retinopathy between insulin-dependent and non-insulin-dependent patients found that insulin usage was associated with retinopathy events (OR 1.36; 95 % CI 1.07–1.73). (El-Asrar, 1999) Similar results were reported in another local study (OR 2.75; 95 % CI 1.61–4.71). (Ahmed et al., 2016) In our study, a significant increase in retinopathy was detected in diabetic patients managed by insulin therapy. Retinopathy was detected in 125 out of 366 patients with HbA1C of 9 % or more, 107 of whom were using insulin therapy, whereas 18 were not, with a p-value of 0.005. Since the DDP-4 inhibitor class was linked with an increased incidence of retinopathy in several in vitro and clinical studies, (Lee et al., 2016, Kang et al., 2021, Kim et al., 2018, Gyu Chul Oh, You-Jeong Ki, Kyung Woo Park, Kyung-Do Han, Hyo-Soo Kim. Association Between Dipeptidyl Peptidase-4 Inhibitors and Diabetic Retinopathy in Korean Patients With Type 2 Diabetes Mellitus: Nationwide Population-Based Cohort Study, 17 August, 2021) this was taken into consideration. Utilizing the Chi-square test, subgroup analysis of our patients who developed retinopathy reveals that there is not enough evidence to conclude the association between retinopathy and the incidence of retinopathy complications. However, our data did not specify the type of retinopathy.

Numerous studies have provided evidence supporting the effectiveness of intensive insulin therapy in postponing the onset and progression of diabetic nephropathy among type 1 and type 2 diabetic individuals. (Diabetes Control and Complications Trial Research Group; Nathan DM, Genuth S, Lachin J, Cleary P, Crofford O, Davis M, Rand L, Siebert C. The effect of intensive treatment of diabetes on the development and progression of long-term complications in insulin-dependent diabetes mellitus. N Engl J Med., 1993, Shichiri et al., 2000, Feldt-Rasmussen et al., 1991, Ohkubo et al., 1995) However, nephropathy was detected in 47 patients, 41 of whom were on insulin therapy in our study population. Our nephropathy findings could be interpreted by having more controlled patients among the non-insulin-managed group since the average HbA1C reduction among diabetic patients with HbA1C of 9 % or more was significantly different between the two groups. Among the insulin therapy group, a total of 286 patients, the average HbA1C was 9.64 %, whereas in the non-insulin therapy group, a total of 80 patients, the average HbA1C was 9.11 % with a p-value of 0.005. On the other hand, neuropathy complications were found in 28 patients, all of whom were on insulin therapy. However, a major limitation of detecting complications is the incomplete documentation in the patients’ electronic medical records.

The inherent limitations of a retrospective observational study include the selection and uneven distribution of comparable groups, lack of homogeneity by enrolling patients in different years, and missing or incomplete documentation. However, it is difficult to find an equal number of patients with uncontrolled type-2 DM with HbA1C>9 on insulin or non-insulin therapy in the real world. Additionally, such a study may be difficult to perform prospectively due to clinician preference for particular anti-diabetic agents. To overcome the lack of homogeneity, we included patients who were followed up within the hospital for at least 5 years. Another limitation of our study is the inability to control confounders between the two groups. These confounders are mostly subjective, such as physical activity habits, nutritional therapy, smoking status, smoking cessation counseling, and psychosocial care.

## 5 Conclusion

In conclusion, our study didn’t find a significant association between the use and cardiovascular complications. In line with clinical practice guidelines, insulin could be introduced as a choice for type-2 diabetic patients with HbA1C≥9. However, insulin therapy could influence the development of other diabetes complications. The association between insulin therapy and the development of other diabetes complications may necessitate further trials.

## CRediT authorship contribution statement

**Sultan Alghadeer:** Writing – review & editing, Validation, Supervision, Resources, Project administration, Methodology, Conceptualization. **Abdullah M. Mubarak:** Methodology, Formal analysis, Data curation. **Bashayr Alsuwayni:** Writing – review & editing, Investigation. **Faisal Almurdhi:** Writing – original draft, Resources, Investigation. **Hazim Almalki:** Writing – original draft, Investigation. **Metib Alotaibi:** Visualization, Validation, Supervision, Conceptualization.

## Declaration of competing interest

The authors declare that they have no known competing financial interests or personal relationships that could have appeared to influence the work reported in this paper.

## References

[b0005] Ahmed R.A., Khalil S.N., Al-Qahtani M.A. (2016). Diabetic retinopathy and the associated risk factors in diabetes type 2 patients in Abha. Saudi Arabia. J Family Community Med..

[b0010] Al Dawish M.A., Robert A.A., Braham R., Al Hayek A.A., Al Saeed A., Ahmed R.A., Al Sabaan F.S. (2016). Diabetes Mellitus in Saudi Arabia: A Review of the Recent Literature. Curr Diabetes Rev..

[b0020] American Diabetes Association (2018). Standards of Medical Care in Diabetes-2018 Abridged for Primary Care Providers. Clin Diabetes..

[b0025] Banday M.Z., Sameer A.S., Nissar S. (2020). Pathophysiology of diabetes: An overview. Avicenna J Med..

[b0030] Brown J.B., Nichols G.A., Perry A. (2004). The burden of treatment failure in type 2 diabetes. Diabetes Care..

[b0035] Chaggar P.S., Shaw S.M., Williams S.G. (2009). Review article: Thiazolidinediones and heart failure. Diab Vasc Dis Res..

[b0040] Cosmi F., Shen L., Magnoli M., Abraham W.T., Anand I.S., Cleland J.G., Cohn J.N., Cosmi D., De Berardis G., Dickstein K., Franzosi M.G., Gullestad L., Jhund P.S., Kjekshus J., Køber L., Lepore V., Lucisano G., Maggioni A.P., Masson S., McMurray J.J.V., Nicolucci A., Petrarolo V., Robusto F., Staszewsky L., Tavazzi L., Teli R., Tognoni G., Wikstrand J., Latini R. (2018). Treatment with insulin is associated with worse outcome in patients with chronic heart failure and diabetes. Eur J Heart Fail..

[b0045] Dal Canto E., Ceriello A., Rydén L., Ferrini M., Hansen T.B., Schnell O., Standl E., Beulens J.W. (2019). Diabetes as a cardiovascular risk factor: An overview of global trends of macro and micro vascular complications. Eur J Prev Cardiol..

[b0050] Davies MJ, Aroda VR, Collins BS, Gabbay RA, Green J, Maruthur NM, Rosas SE, Del Prato S, Mathieu C, Mingrone G, Rossing P, Tankova T, Tsapas A, Buse JB. Management of Hyperglycemia in Type 2 Diabetes, 2022. A Consensus Report by the (ADA) and the (EASD). Diabetes Care. 2022; 45(11):. 10.2337/dci22-0034 PMC10008140 36148880

[b0055] Dean L, McEntyre J. The Genetic Landscape of Diabetes [Internet]. Bethesda (MD): National Center for Biotechnology Information (US); 2004. Chapter 1, Introduction to Diabetes. 2004 Jul 7. Available from: https://www.ncbi.nlm.nih.gov/books/NBK1671/.

[b0060] Diabetes Control and Complications Trial Research Group; Nathan DM, Genuth S, Lachin J, Cleary P, Crofford O, Davis M, Rand L, Siebert C. The effect of intensive treatment of diabetes on the development and progression of long-term complications in insulin-dependent diabetes mellitus. N Engl J Med. 1993;329(14):977-86. 10.1056/NEJM199309303291401 8366922

[b0065] El-Asrar AM, Al-Rubeaan KA, Al-Amro SA, Kangave D, Moharram OA. Risk factors for diabetic retinopathy among Saudi diabetics. Int Ophthalmol. 1998-1999;22(3):155-61. 10.1023/a:1006240928938 10548460

[b0070] Feldt-Rasmussen B., Mathiesen E.R., Jensen T., Lauritzen T., Deckert T. (1991). Effect of improved metabolic control on loss of kidney function in type 1 (insulin-dependent) diabetic patients: an update of the Steno studies. Diabetologia..

[b0075] Garg R., Chaudhuri A., Munschauer F., Dandona P. (2006). Hyperglycemia, insulin, and acute ischemic stroke: a mechanistic justification for a trial of insulin infusion therapy. Stroke..

[b0080] Gyu Chul Oh, You-Jeong Ki, Kyung Woo Park, Kyung-Do Han, Hyo-Soo Kim. Association Between Dipeptidyl Peptidase-4 Inhibitors and Diabetic Retinopathy in Korean Patients With Type 2 Diabetes Mellitus: Nationwide Population-Based Cohort Study, 17 August 2021, PREPRINT (Version 1) available at Research Square [https://doi.org/10.21203/rs.3.rs-666003/v1].

[b0085] Houlden R., Ross S., Harris S., Yale J.F., Sauriol L., Gerstein H.C. (2007). Treatment satisfaction and quality of life using an early insulinization strategy with insulin glargine compared to an adjusted oral therapy in the management of type 2 diabetes: The Canadian INSIGHT study. Diabetes Res Clin Pract..

[b0090] Kang E.Y., Kang C., Wu W.C., Sun C.C., Chen K.J., Lai C.C., Chen T.H., Hwang Y.S. (2021). Association between Add-On Dipeptidyl Peptidase-4 Inhibitor Therapy and Diabetic Retinopathy Progression. J Clin Med..

[b0095] Kim N.H., Choi J., Kim N.H., Choi K.M., Baik S.H., Lee J., Kim S.G. (2018). Dipeptidyl peptidase-4 inhibitor use and risk of diabetic retinopathy: A population-based study. Diabetes Metab..

[b0100] Laiteerapong N., Ham S.A., Gao Y., Moffet H.H., Liu J.Y., Huang E.S., Karter A.J. (2019). The Legacy Effect in Type 2 Diabetes: Impact of Early Glycemic Control on Future Complications (The Diabetes & Aging Study). Diabetes Care..

[b0105] Lee C.S., Kim Y.G., Cho H.J., Park J., Jeong H., Lee S.E., Lee S.P., Kang H.J., Kim H.S. (2016). Dipeptidyl Peptidase-4 Inhibitor Increases Vascular Leakage in Retina through VE-cadherin Phosphorylation. Sci Rep..

[b0110] Nakar S., Yitzhaki G., Rosenberg R., Vinker S. (2007). Transition to insulin in type 2 diabetes: family physicians' misconception of patients' fears contributes to existing barriers. J Diabetes Complications..

[b0115] Nathan DM, Buse JB, Davidson MB, Ferrannini E, HolmanRR, Sherwin R, Zinman B. Medical management of hyperglycemia in type 2 diabetes: a consensus algorithm for the initiation and adjustment of therapy: a consensus statement of the American Diabetes Association and the European Association for the Study of Diabetes. Diabetes Care. 2009; 32:193 – 203. 10.2337/dc08-9025 PMC2606813 18945920

[b0120] Ohkubo Y., Kishikawa H., Araki E., Miyata T., Isami S., Motoyoshi S., Kojima Y., Furuyoshi N., Shichiri M. (1995). Intensive insulin therapy prevents the progression of diabetic microvascular complications in Japanese patients with non-insulin-dependent diabetes mellitus: a randomized prospective 6-year study. Diabetes Res Clin Pract..

[b0125] Polonsky W.H., Fisher L., Guzman S., Villa-Caballero L., Edelman S.V. (2005). Psychological insulin resistance in patients with type 2 diabetes: the scope of the problem. Diabetes Care..

[b0130] Rodbard HW, Cariou B, Zinman B, Handelsman Y, Philis-Tsimikas A, Skjøth TV, Rana A, Mathieu C; BEGIN Once Long trial investigators. Comparison of insulin degludec with insulin glargine in insulin-naive subjects with Type 2 diabetes: a 2-year randomized, treat-to-target trial. Diabet Med. 2013;30(11):1298-304. 10.1111/dme.12303 PMC4208679 23952326

[b0135] Ruige J.B., Assendelft W.J., Dekker J.M., Kostense P.J., Heine R.J., Bouter L.M. (1998). Insulin and risk of cardiovascular disease: a meta-analysis. Circulation..

[b0140] Schwartz S.S., Jellinger P.S., Herman M.E. (2016). Obviating much of the need for insulin therapy in type 2 diabetes mellitus: A re-assessment of insulin therapy's safety profile. Postgrad Med..

[b0145] Shichiri M., Kishikawa H., Ohkubo Y., Wake N. (2000). Long-term results of the Kumamoto Study on optimal diabetes control in type 2 diabetic patients. Diabetes Care..

[b0150] Swinnen S.G., Hoekstra J.B., DeVries J.H. (2009). Insulin therapy for type 2 diabetes. Diabetes Care..

[b0155] Weng J., Li Y., Xu W., Shi L., Zhang Q., Zhu D., Hu Y., Zhou Z., Yan X., Tian H., Ran X., Luo Z., Xian J., Yan L., Li F., Zeng L., Chen Y., Yang L., Yan S., Liu J., Li M., Fu Z., Cheng H. (2008). Effect of intensive insulin therapy on beta-cell function and glycaemic control in patients with newly diagnosed type 2 diabetes: a multicentre randomised parallel-group trial. Lancet..

